# Biofunctional Textiles: Antioxidant and Antibacterial Finishings of Cotton with Propolis and Honey

**DOI:** 10.3390/ijms25158034

**Published:** 2024-07-23

**Authors:** Ana Sofia Freitas, Rui Oliveira, Alice Ribeiro, Cristina Almeida-Aguiar

**Affiliations:** 1 CITAB—Centre for the Research and Technology of Agro-Environmental and Biological Sciences, University of Minho, Campus de Gualtar, 4710-057 Braga, Portugal; anasofiapfreitas@gmail.com; 2 Department of Biology, School of Sciences, University of Minho, Campus de Gualtar, 4710-057 Braga, Portugal; ruipso@bio.uminho.pt; 3 CBMA—Centre of Molecular and Environmental Biology, University of Minho, Campus de Gualtar, 4710-057 Braga, Portugal; 4 CeNTI—Centre for Nanotechnology and Smart Materials, 4760-034 Vila Nova de Famalicão, Portugal; alribeiro@centi.pt

**Keywords:** biofunctional textiles, cotton, propolis, honey, antioxidant potential, antibacterial activity, washing fastness

## Abstract

The growing activity in the textile industry has been demanding the search for new and innovative technologies to meet consumers’ needs regarding more sustainable and ecological processes, with functionality receiving more attention. Bee products are known for their wide spectra of properties, including antioxidant and antibacterial activities. Propolis and honey are the most popular and used since ancient times for the most diverse applications due to their health benefits. With the increasing need for safer and more sustainable practices, the use of natural products for the functional finishing process can be a suitable alternative due to their safety and eco-friendly nature. For that, a biosolution, composed of a mixture of propolis and honey in water, was used to perform the functional finishing of cotton knits, both in the presence and in the absence of potassium alum as a chemical mordant. The fastness strength was also evaluated after three washing cycles. The antioxidant potential of the biosolution, assessed with the in vitro ABTS scavenging assay, provided textiles with the capacity to reduce more than 90% of the ABTS radical, regardless of the mordant presence and even after three washing cycles. Furthermore, biofunctional textiles decreased the growth of *Bacillus subtilis*, *Propionibacterium acnes*, *Escherichia coli,* and, particularly, *Staphylococcus aureus* cultures after 24 h of incubation with an increase in antibacterial activity when potassium alum was used. These findings show that bee products are promising and effective alternatives to be used in the textile industry to confer antioxidant and antibacterial properties to cotton textiles, thereby enhancing human health.

## 1. Introduction

Bee products can be used as food, with active products for medicinal and cosmetic purposes or inactive products used as bases [1]. Interest in natural products, such as honey and propolis, has been increasing due to their important role in human health and well-being [2]. Honey, considered a natural food of high nutritional value and used in human consumption for its organoleptic and therapeutic characteristics over centuries [3,4], as well as propolis, both a building and a defensive material of the beehive [5], are the most popular bee products. Both are commercialized in different parts of the world and recognized as important sources of bioactive compounds with properties for several applications [6,7,8]. By 2028, the Global Honey and Propolis Markets are expected to reach a value of USD 17.34 billion, with a compound annual growth rate (CAGR) of 8.2%, and USD 60.9 million, with a CAGR of 4%, respectively [9,10].

The growing demand for natural products relies on the health-conscious awareness of consumers towards the potential toxicity of the chemicals used in conventional personal care products and the benefits of the properties, especially the antimicrobial and antioxidant properties, that natural products are known to own [3]. The global natural and organic cosmetics market is projected to reach USD 54.5 billion by 2027 and is expected to grow at a CAGR of 5.2% between 2018 and 2027, mostly due to the increasing demand for skin and hair products [11]. In 2021, the global natural skin care products market was rated at USD 6.7 billion, growing at a CAGR of 6.6% from 2022 to 2030 [12]. Bee products, especially honey and propolis, are known for their effects on skin care, mostly due to their antimicrobial, anti-inflammatory, antiviral, regenerative, and antiaging properties [13]. Skin, as the largest human organ, represents the first protection barrier against environmental aggressions and is mostly covered by textile [14]. Biofunctional textiles are fabrics with acquired properties which can act like release systems able to deliver substances such as bioactive principles to the skin, having been a subject of interest due to their applicability as biomedical devices for skin treatment or cosmetics [15]. Chemicals used to impart antibacterial activity to textiles are mostly synthetic and usually associated with some level of toxicity, which can be less effective against bacteria due to their extensive use and consequent development of resistance [16]. Natural substances, namely, flavonoids, have already been used to provide textiles with bioactivities [17]; however, their stability and bioavailability are often questioned, making researchers search for alternative methods for textile functionalization, using inclusion complex nanofibers [17,18,19] to improve both the stability and efficiency of the functionalization process, and the bioavailability of the active substances.

The need for the development of new functional products that meet consumers’ increasing concerns with health and well-being leads to the quest for new and sustainable technologies by the textile industry with less environmental impact and supporting a circular economy [20]. Relying on the growing need for safer and sustainable alternatives in textile functionalization, the aim of this work was to investigate the potential of the beehive products, especially propolis and honey, for textile (cotton) functionalization, with antioxidant and antibacterial activities. Additionally, the potassium alum mordant capacity to improve textile’s chemical affinity and functionalization resistance to the washing process was assessed as well, given the importance of reuse for sustainability.

## 2. Results

### 2.1. Antioxidant and Antimicrobial Properties of the Functionalized Textiles

According to our previous work on antioxidant and antimicrobial synergisms between propolis and honey [21], where several mixtures of propolis and honey were tested against a wide panel of bacteria, a mixture of 200 μg mL^−1^ of G18.EE with 5% H18 was able to inhibit the growth of *Bacillus subtilis*, *B*. *megaterium*, *B*. *cereus*, *Staphylococcus aureus,* and *Propionibacterium acnes*, which led to the selection of this mixture for textile functionalization. In order to assess if the selected mixture had the desired antioxidant potential, the in vitro 2,2’-azino-bis(3-ethylbenzothiazoline-6-sulfonic acid (ABTS) scavenging assay was performed before textile functionalization. The mixture exhibited limited antioxidant potential with only a 58.8 ± 1.2% ABTS radical reduction. Therefore, and based on previous results which showed a dilution of the antioxidant potential of the mixture with an increasing honey concentration [21], it was decided to increase by five times the concentration of propolis (from 200 to 1000 ug mL^−1^). As presented in Table 1, this mixture of 1000 μg mL^−1^ G18.EE with 5% H18 showed a higher antioxidant potential (95.9 ± 0.7% ABTS radical reduction); therefore, this mixture was used as the biofunctional solution (BS) for textile functionalization.

The antioxidant potential of the biofunctional textiles assessed by the in vitro ABTS scavenging assay [22] shows the control sample, cotton (CO) only, with a significantly lower antioxidant capacity value (27.4 ± 1.3% ABTS radical reduction) when compared to the functionalized samples (> 90% ABTS radical reduction; *p* < 0.001; Table 1). The functionalization process did not lead to antioxidant activity loss, as BS and both CO + BS and CO + BS_PA showed no significant differences in their capability to reduce the ABTS radical.

The limitations of natural dyeing processes such as the lack of uniformity, low yield, poor adhesion, and poor fastness properties [23] can be overcome with the use of mordants [24], which allow us to increase the biocompound affinity and to enhance the fastness of the functionalized textiles [25]. To assess if the antioxidant potential of the biofunctional textiles can be potentiated using a mordant, one textile knit was treated simultaneously with the biosolution and potassium alum (CO + BS_PA) and tested by the in vitro ABTS scavenging assay. The use of potassium alum as the mordant did not show improvements in the antioxidant capacity as there were no significant differences when compared to the textiles treated with the biosolution alone (98.5 ± 0.2% or 98.4 ± 0.4% ABTS radical reduction in the presence or in the absence of potassium alum, respectively; Table 1). 

Unlike those observed for the antioxidant potential, there were significant differences between the antibacterial activity of CO + BS and CO + BS_PA, with the latter being more effective in decreasing the growth of *B. subtilis*, *P. acnes*, and *Escherichia coli* (Table 2). These results highlight the importance of using a mordant to enhance the antibacterial activity of the biofunctional textile, although *S. aureus* was almost equally affected by both CO + BS and CO + BS_PA. Nevertheless, in the absence of a mordant, there were significant differences between the ability to decrease bacterial growth: *S. aureus* was the most affected (94.8 ± 0.8% growth reduction), followed by *B. subtilis*, *E. coli*, and, lastly, *P. acnes*, the least affected strain (12.3 ± 7.1% growth reduction; Table 2). But, when potassium alum was used, no significant differences were detected between the ability to decrease the growth of all the tested strains (Table 2). Potassium alum, an environmentally friendly compound with no harmful effects on ecosystems and approved for medical use [26], besides improving the antibacterial activity of textiles, may also help to decrease the existing variability between the strains’ susceptibility to the treatment.

### 2.2. Strength of Textile Functionalization to Washing

Functionalized clothing is meant to be worn and washed multiple times. Therefore, it is important to assess the maintenance of textiles’ functionality upon cycles of washing. Hence, the antioxidant potential of the biofunctional textiles was evaluated after three washing cycles (3WC; Table 3). The control sample (CO_3WC_; 26.5 ± 2.0% ABTS radical reduction), similarly to the control sample before the washing process (CO; 27.4 ± 1.3% ABTS radical reduction; Table 1), showed a significantly lower antioxidant capacity than the treated samples (> 90% ABTS radical reduction; *p* < 0.001). Likewise, the use of potassium alum as a mordant did not show an improvement in antioxidant capacity as there were no significant differences when comparing the BS-treated textiles after three washing cycles (93.7 ± 0.6 and 91.8 ± 1.9% ABTS radical reduction for CO + BS_3WC_ and CO + BS_PA_3WC_, respectively). Despite the slight decrease in the antioxidant capacity of the biofunctional textiles after three washing cycles (CO + BS_3WC_ and CO + BS_PA_3WC_; Table 3) comparatively to the samples before the washing process (CO + BS and CO + BS_PA; Table 1), all samples showed a percentage of ABTS radical reduction above 90%.

**Figure 1:**
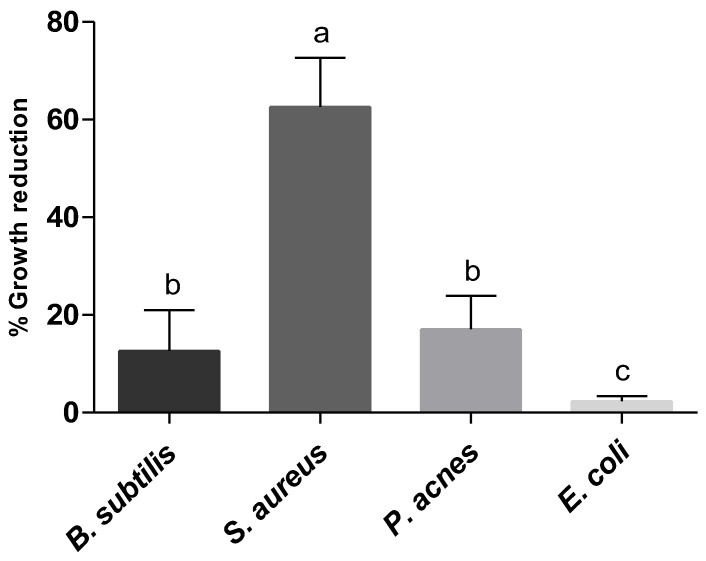
Assessment of the washing process influence on the antibacterial activity of the textiles against four bacterial strains: *Bacillus subtilis*, *Staphylococcus aureus*, *Escherichia coli,* and *Propionibacterium acnes*. Strains in mid-exponential phase were transferred to Erlenmeyers with LB with the respective textile (see Section 4 for details). Samples tested were cotton (CO) and cotton after three washing cycles (CO_3WC_). After 24 h incubation at 37 °C, antibacterial activity was determined upon colony-forming units count and results were expressed in growth reduction percentage relative to control (CO) and respective standard deviation (SD). Statistical analysis was performed by one-way ANOVA, followed by Tukey’s test for significance. Different letters mean statistically significant (*p* < 0.05) differences between mean values.

Beyond the antioxidant potential maintenance, the antibacterial activity must also persist after the washing process. To assess the influence of the washing process on textiles’ antibacterial activity, the growth reduction percentage of the control after the three washing cycles (CO_3wc_) was expressed relatively to the control without any washing (CO). As shown in Figure 1, washed cotton affected bacteria differently, with *S*. *aureus* being the most affected one (63.7 ± 10.0% growth reduction), followed by *B*. *subtilis* and *P*. *acnes* being equally affected, and *E*. *coli* being the least affected bacterium (2.3 ± 1.3% growth reduction) by the washing process. These results suggest that the detergent itself possesses antibacterial activity, affecting all the tested strains, albeit differently. Thus, the detergent selection is a determining factor as it could influence the antibacterial activity of the textile after the washing process in a dimension that will depend on the target strain.

The treated textiles did not lose their function and maintained their antibacterial activity after three washing cycles, except against *E*. *coli* (Table 2 and Table 4). Without the use of the mordant, the differences between the ability to decrease the growth of the tested strains were less marked than before the washing process (Table 4), with *S*. *aureus* being the most affected (86.1 ± 9.8% growth reduction), followed by *P*. *acnes* (56.9 ± 9.4% growth reduction), *B*. *subtilis* (47.1 ± 3.5% growth reduction), and *E*. *coli* (43.6 ± 0.9% growth reduction) being the least affected species. The use of potassium alum seems to be less significant in the case of *S*. *aureus* and *P*. *acnes*, or even a disadvantage in the case of *E*. *coli*. Nevertheless, its use increased the antibacterial activity against *B*. *subtilis* by double (from 47.1 ± 3.5% to 99.8 ± 0.01% growth reduction; *p* < 0.001; Table 4). Biofunctional textiles, either with or without the simultaneous treatment with potassium alum, exert distinct effects depending on the strain tested, meaning that, depending on the application, several assays with different mordants and several washing cycles are needed to ensure the intended effect on the textile.

## 3. Discussion

Several studies have been showing that natural products, in addition to coloring textiles, give them particular functional properties such as antioxidant and antimicrobial activities [27,28,29,30,31,32,33,34,35,36,37,38,39,40,41,42,43]. However, just a few authors have investigated the potential of propolis as a functional finishing agent to be applied in the textile industry [27,28,34,35,36,37,38]. According to our knowledge, this study is the first to explore the combined capability of propolis and honey in providing smart textiles with antioxidant and antibacterial activities and the first one to test the action of biofunctional textiles against *P*. *acnes*, the most abundant bacterial species found on human skin and involved in the development of various inflammatory skin conditions, like acne [39].

Moreover, to the best of our knowledge, the investigation on the antioxidant potential of textiles dyed with propolis is lacking. Within our investigation group, pioneers in the matter, the great antioxidant potential of cotton dyed with propolis from Gerês (88.7 ± 0.4% ABTS radical reduction) was shown [38]. In the present study, we used 10 times less the amount of propolis used by Cardoso et al. (2021) [38], but in combination with 5% of honey, achieving a higher antioxidant capacity (98.4 ± 0.4 and 98.5 ± 0.2% ABTS radical reduction for CO + BS and CO + BS_PA, respectively; Table 1).

The direct contact of textile with skin, a habitat to millions of micro-organisms, can lead to dermal infection, allergies, unpleasant odor, and damage to the textile properties due to the proliferation of pathogenic micro-organisms, mainly bacteria, such as *S*. *aureus*, *Pseudomonas aeruginosa,* and *E. coli* [40,41]. Therefore, the antibacterial activity of textile, particularly that provided by natural agents, is an important parameter to consider for textiles which are in direct contact with skin. For the first time, the antibacterial effect of propolis-dyed cotton was investigated against *S*. *aureus* and *E*. *coli* [28]. The finishing of the textile was carried out using different mordants, such as glyoxal, arcofix, glutraldehyde, and 1,2,3,4 butanetetracarboxylic acid (BTCA) for propolis fixation, showing the importance of the use of a crosslinking agent to achieve a higher interaction between propolis and the textile surface and to consequently increase the antibacterial activity. Unlike our results, the untreated cotton showed no differences when compared to the propolis-treated cotton [28]. In our study, the BS treatment itself provided substantial antibacterial activity when compared to the untreated cotton sample. The antibacterial activity of cotton treated with Romanian propolis was evaluated against *S. aureus, E. coli*, *P. aeruginosa,* and *Streptococcus* β haemolytic, showing higher activity against *S*. *aureus* and *E*. *coli* [27]. The activity was enhanced with a simultaneous treatment with chitosan, a natural polysaccharide widely used as a finishing agent for surface modification due to its antimicrobial activity and eco-friendly nature [42].

The use of bee products such as propolis, honey, and beeswax as natural antibiotics in the medical fields by providing antibacterial activity to non-woven fabrics like cotton gauze has proven its effectiveness against *S*. *aureus*, *E*. *coli,* and *Klebsiella pneumoniae* with or without a crosslinking agent such as chitosan [31,35,37,43]. Propolis in combination with nanostructures such as polylactic acid (PLA; a plant-derived thermoplastic) and polyvinyl alcohol (PVA; a water-soluble synthetic polymer) was found to be active against bacteria by inhibiting *S*. *aureus* proliferation, revealing the potential to be used in medical textiles for burns, mouth ulcers, and human diabetes wounds [34,36]. Manuka honey was found to totally reduce the growth of *S*. *aureus* and *K*. *pneumonieae* [44]. Moreover, honey incorporated into an alginate hydrogel was found to be a promising solution for wound dressing, having demonstrated a high antioxidant capacity and antibacterial activity against the Gram-positive *S*. *aureus* and the Gram-negative *E*. *coli* [45].

The fastness to the washing process was also evaluated, showing the excellent durability of the treatments to the washing process up to twenty washing cycles and three washing cycles, respectively [28,38]. The importance of using potassium alum (pre-treatment) was demonstrated as well, after five washing cycles, by preventing the antioxidant activity decrease shown by the sample treated only with propolis (from 88.7 ± 0.4 to 62.8 ± 8.7% ABTS radical reduction after five washing cycles) [38]. In the present study, the washing fastness was evaluated for three washing cycles, and no loss of function was registered for the textiles. Potassium alum itself is found to possess antimicrobial activity against *S*. *aureus* and *E*. *coli*, in a concentration-dependent manner [46], with its activity mainly attributed to its ability to cause the disruption of the bacterial cell wall, leading to cell lysis and, consequently, death, and also by creating an acidic environment that is unsuitable for bacteria, causing the precipitation of proteins that are important for bacterial function [47]. On the other hand, the simultaneous treatment with potassium alum did not have a significant impact on the antioxidant and antimicrobial properties of the functionalized textiles (Table 4). This is a significant outcome considering the additional costs and negative impacts of the use of chemicals by the textile industry.

In short, biofunctional textiles with the propolis and honey mixture showed antioxidant and antibacterial activity even after three washing cycles, which is important for the lifespan of the textile. The prevalence of these activities after textile washing helps to ensure the durability of the functionalization, consequently reducing the waste. It would be interesting to test the textile bioactivities when functionalized with propolis and honey, individually, for comparison reasons. However, we did not consider that for this work because this combination of propolis and honey was hypothesized to yield the most impactful results based on our preliminary studies [21]. In fact, we demonstrated that the synergistic effects of honey and propolis not only enhance their individual properties but also allow the use of smaller amounts of propolis, which is particularly beneficial considering the relatively lower production rates of propolis compared to honey. In general, the use of potassium alum as a chemical mordant was shown to be advantageous by maintaining or improving the overall antibacterial activity of the textiles with more or less impact depending on the tested strain, with the exception of *E*. *coli,* where such use was revealed to be a disadvantage after the washing process. Nevertheless, the assessment of the potential of other mordants would be an asset for the investigation. Aligned with these points, the comfort properties of the textile after the treatments would also be an important aspect to evaluate once that could be negatively affected by the treatment and, consequently, change the consumer experience.

Such textiles, being in direct contact with the skin, can provide the continuous delivery of active compounds, which can cause allergic reactions, especially for those with sensitive skin; thus, it is also important to analyze the textile biocompatibility with the skin. Additionally, to address potential allergenic concerns, there are several approaches we can consider to ensure both efficacy and safety: dose–response studies, finding an optimal concentration range maximizing the antimicrobial activity while the minimizing allergenic potential; and controlled release mechanisms, as advanced encapsulation techniques, allowing a minimal initial contact with skin while gradually releasing active compounds over time, and maintaining the antimicrobial efficacy.

Given the inherent diversity present in natural products such as propolis and honey, and the implications that this particularity has on their standardization, the evaluation of quality criteria for samples, as the analysis of the chemical composition, is imperative in any scientific investigation [48,49,50,51]. Indeed, some previous studies have linked the presence of specific compounds with propolis antioxidant activity such as gallic acid, HHDP-hexoside, digalloyl hexoside, gallotannin, tannins, apigenin, kaempferol, galangin, caffeic acid derivatives, and coumaric acid derivatives [52,53]. Other compounds such as ellagic acid, ferulic acid, pinobanksin-5-methyl-ether, quercetin-3-methyl ether, kaempferol, isorhamnetin, acacetin, kaempferide, kaempferol-methoxy-methyl ether, and caffeic acid derivatives have been linked with propolis antimicrobial activity [53,54,55]. Some of these compounds, namely, gallic acid, caffeic acid, kaempferol, acacetin, ferulic acid, and galangin, have been identified in G18.EE [53]. The main compounds responsible for honey’s antimicrobial activity include phenolic acids such as cinnamic acids and their esters, and flavonoids such as hesperetin and rutin [56,57,58]. Other compounds such ascorbic acid, flavonoids, carotenoids, and phenolic acids have been linked to honey’s antioxidant potential [57,58,59]. Future work will focus on natural products’ quality by an assessment of the chemical composition through comprehensive analyses. Driven by the promising results obtained so far, a chemical analysis will allow the standardization of propolis and honey, ensuring consistency, efficacy, and safety in their application.

## 4. Materials and Methods

### 4.1. Propolis and Honey Sample and Extract Preparation

Propolis and honey samples used in this work were harvested from an apiary located near the Cávado River, in a protected area of the Peneda-Gerês National Park, in the north of Portugal (41°45′41.62″ N; 7°58′03.34″ W), and kindly provided by the beekeeper. Both propolis and honey samples were collected in 2018 and coded as G18 (Gerês 2018) and H18 (Honey 2018), respectively.

Honey was diluted to a stock solution of 50% (*v*/*v*) in sterile water—H18_50_. Propolis was extracted with absolute ethanol as previously described [21]. Briefly, propolis was extracted twice with absolute ethanol (80 mL and 50 mL, respectively) followed by filtration under vacuum. The resulting filtrates were pooled and dried in a Buchi Rotavapor RE 121 (40 rpm, at 40 °C), yielding the ethanol extract (EE) G18.EE.

### 4.2. Textile Functionalization

#### 4.2.1. Preparation of Biofunctional Solution

Biofunctional solution was prepared by mixing G18.EE and H18_50_ with water to a final concentration of 1000 µg mL^−1^ and 5% (*v*/*v*), respectively. The solutions were stored at 4 °C, in the dark, until further use.

#### 4.2.2. Textile Treatments

For textile functionalization, 100% CO was chosen, and the adopted technology was impregnation by exhaustion in a thermostatic bath (Grant OLS200, Grant Instruments (Cambridge) Ltd, United Kingdom). Two CO textile knits were rinsed with water and placed into different tubes. One tube was filled with the BS while the second tube received an identical volume of BS with the chemical mordant (BS_PA), which was prepared with 10 g/L of PA (KAl(SO_4_)·12H_2_O). A ratio of 1:20 (g/mL) textile/BS was used in the bath and incubation was performed at 40 °C and 100 rpm for 90 min. After textile functionalization, the knits CO (used as control without any treatment), CO + BS (cotton knit treated with BS), and CO + BS_PA (cotton knit cotreated with BS and PA) were dried for 24 h at room temperature.

### 4.3. Strength of Textile Functionalization to Washing

Biofunctional solution-treated textiles were washed to evaluate their washing fastness. For that, CO, used as control, and the biofunctional knits CO + BS and CO + BS_PA were divided in two equal parts, one part of each being subject to 3WC in a domestic washing machine (Indesit IWDC 6105, Indesit Company S.p.A., Fabriano, Italy). Each washing cycle was performed at 30 °C for 1 h with addition of SoflanTM (detergent suitable for less abrasive washes; Colgate-Palmolive Company, New York, NY, USA). After the three washing cycles, textile samples—CO_3WC_, CO + BS_3WC_, and CO + BS_PA_3WC_—were dried at room temperature and assessed for antioxidant and antibacterial activities.

### 4.4. In Vitro Evaluation of the Antioxidant Potential

ABTS radical scavenging assay was used for in vitro evaluation of the antioxidant potential [22], with slight modifications adopted according to the samples (BS and BS_PA or textiles) under evaluation. Firstly, an aqueous solution of ABTS (7 mM; Sigma-Aldrich, Lisbon, Portugal) and potassium persulfate (2.45 mM; Sigma-Aldrich) was kept in the dark, at room temperature for 16 h, and then diluted in phosphate buffer 0.1 M (pH 7.4) until an optical density of 0.7 ± 0.025 (PerkinElmer Lambda 35, Shelton, CT, USA) was reached at 734 nm (OD_734_). This ABTS solution was used as working solution.

Antioxidant potential of the biofunctional solution was evaluated by adding 30 µL of the BS to 2970 µL of the ABTS working solution (using a 1:99 proportion in the reaction mixture), followed by 6 min incubation at room temperature. The antioxidant potential of textiles, either functionalized (CO + BS, CO + BS_PA, CO + BS_3WC_, and CO + BS_PA_3WC_) or non-functionalized (CO and CO_3WC_), was evaluated by adding 3 mL of ABTS working solution to 0.075 g of the different sample knits, followed by 30 min incubation at room temperature. L-cysteine (0.3 g L^−1^) was used as positive control. Absorbance of the reaction was measured at 734 nm and results were expressed in percentage of ABTS radical reduction according to the following equation:

% inhibition = [(A_control_ − A_sample_) × 100]/A_control_

where A_sample_ is the absorbance of the samples and A_control_ the absorbance of the control (ABTS working solution and ethanol, water, or a mixture of both, the solvents of G18.EE, H18_50_, or BS, respectively, and only ABTS for the textile samples), after 6 or 30 min of reaction, depending on the tested samples.

### 4.5. Determination of Antibacterial Properties

Antibacterial activity was evaluated against the Gram-negative bacterium *Escherichia coli* CECT 423 and the Gram-positive bacteria *Bacillus subtilis* 48886, *Staphylococcus aureus* ATCC 6538 (MSSA), and *Propionibacterium acnes* H60803, obtained from the microbial collection of the Department of Biology of the University of Minho. Bacteria were cultured in LB (Luria–Bertani—0.5% *w*/*v* yeast extract, 1% *w*/*v* tryptone, and 1% *w*/*v* NaCl) or in solid medium (LBA by adding 2% *w*/*v* agar to the LB recipe). Growth was performed at 200 rpm and 37 °C and monitored by optical density measurement at 600 nm (OD_600_).

Antibacterial activity of BS was assessed by the agar dilution method [60]. Briefly, 5 µL drops of exponentially growing bacterial cultures were transferred onto LBA plates supplemented with 1000 μg mL^−1^ G18.EE and 5% H18. Control was prepared with LBA containing the solvents used in the same volumes as the sample. Plates were incubated at 37 °C for 24 h. Bacterial susceptibility was determined by observing the presence or absence of growth.

The antibacterial activity of the textiles, either biofunctionalized or non-functionalized, was evaluated according to Nobre (2018) [61] with slight modifications as follows: Firstly, textiles were cut into pieces of 0.1 g and sterilized under UV light for 10 min each side. To prepare the cells for the experiments, overnight microbial cultures were diluted with the appropriated fresh medium to an OD_600_ of 0.05 in 10 mL. A sample of each textile (CO, CO + BS, CO + BS_PA, CO_3WC_, CO + BS_3WC_, and CO + BS_PA_3WC_) was added to the culture and incubated at 200 rpm, at 37 °C, for 24 h. The antibiotic ampicillin (200 µg mL^−1^) was used as positive control. Each bacterial suspension was collected, serially diluted from 10^−1^ to 10^−7^, and drops of 40 µL of diluted microbial cultures were transferred onto LBA plates. After 24 h incubation at 37 °C, colonies were counted and results expressed in growth reduction percentage relative to controls (CO or CO_3WC_), according to the following equation:

% reduction = [(B − A)/B] × 100

where A is the number of colony-forming units in the biofunctionalized textiles and B is the number of colony-forming units in the non-functionalized textile (CO or CO_3WC_), after 24 h incubation.

### 4.6. Statistical Analysis

Experiments were carried out in triplicate and results were expressed as mean ± standard deviation (SD). One-way ANOVA followed by Tukey’s test for multiple comparisons were used to assess treatment effect. Differences considered statistically significant (*p* < 0.05) were distinguished with different letters.

## 5. Conclusions

Synthetic chemicals commonly used in the textile dyeing process are usually associated with adverse side effects to the environment and health. Global demand for safe, effective, and natural products has been increasing in parallel with consumers’ concerns about personal and environmental health. Beehive products, known for their complex chemical composition linked with their wide range of bioactivities, have shown great potential to be used by the textile industry as natural dyeing products with the ability to improve the performance of textiles for specific purposes such as antioxidant and antibacterial finishes. In this sense, beehive products, such as propolis and honey, proved to be promising natural products to be introduced in human daily personal care, for instance, in face masks due to their antioxidant and antimicrobial effects, with the possibility of several reuses.

## Figures and Tables

**Table 1:** Antioxidant potential of the biofunctional solution (BS) and textiles measured by the in vitro ABTS scavenging assay expressed as % ABTS radical reduction and respective standard deviation (SD). Samples tested were cotton (CO), cotton treated with a biosolution (CO + BS) composed by a mixture of propolis (1000 µg mL^−1^) and honey (5%) in water, and cotton treated simultaneously with BS and potassium alum (CO + BS_PA). L-cysteine (0.3 g L^−1^) was used as positive control. Statistical analysis was performed by one-way ANOVA, followed by Tukey’s test for significance. Different letters mean statistically significant (*p* < 0.05) differences between mean values.

Textile Samples	ABTS Scavenging Activity
	% ABTS Radical Reduction
CO	27.4 ± 1.3 ^a^
CO + BS	98.4 ± 0.4 ^b^
CO + BS_PA	98.5 ± 0.2 ^b^
Biofunctional Solution (BS)	95.9 ± 0.7 ^b^

**Table 2:** Antibacterial activity of the biofunctional textiles, assessed by colony-forming units, against four bacterial strains: *Bacillus subtilis*, *Staphylococcus aureus*, *Escherichia coli,* and *Propionibacterium acnes*. Strains in mid-exponential phase were transferred to Erlenmeyers with LB with the respective textile (see Section 4 for details). Samples tested were cotton (CO), cotton treated with a biosolution (CO + BS) composed by a mixture of propolis (1000 µg mL^−1^) and honey (5%) in water, and cotton treated simultaneously with the BS and potassium alum (CO + BS_PA). After 24 h incubation at 37 °C, antibacterial activity was determined upon colony-forming units count and results were expressed in growth reduction percentage relative to control (CO) and respective standard deviation (SD). Statistical analysis was performed by one-way ANOVA, followed by Tukey’s test for significance. Different letters mean statistically significant (*p* < 0.05) differences between mean values.

Strains	Antibacterial Activity % Growth Reduction	
	CO + BS	CO + BS_PA
Gram-positive bacteria		
*Bacillus subtilis*	55.1 ± 18.1 ^c^	88.2 ± 17.7 ^a,b^
*Staphylococcus aureus*	94.8 ± 0.8 ^a^	83.9 ± 2.1 ^a,b^
*Propionibacterium acnes*	12.3 ± 7.1 ^d^	78.5 ± 9.8 ^b^
Gram-negative bacterium		
*Escherichia coli*	21.7 ± 3.2 ^d^	66.6 ± 0.6 ^c,b^

**Table 3:** Antioxidant potential of the textiles after three washing cycles (3WC) measured by the in vitro ABTS scavenging assay and expressed as % ABTS radical reduction and respective standard deviation (SD). Samples tested were cotton (CO_3WC_), cotton treated with a biosolution (CO + BS_3WC_) composed by a mixture of propolis (1000 µg mL^−1^) and honey (5%) in water, and cotton treated simultaneously with BS and potassium alum after three washing cycles (CO + BS_PA_3WC_). L-cysteine (0.3 g L^−1^) was used as positive control. Statistical analysis was performed by one-way ANOVA, followed by Tukey’s test for significance. Different letters mean statistically significant (*p* < 0.05) differences between mean values.

Textile Samples	ABTS Scavenging Activity
	% ABTS Radical Reduction
CO_3WC_	26.5 ± 2.0 ^a^
CO + BS_3WC_	93.7 ± 0.6 ^b^
CO + BS_PA_3WC_	91.8 ± 1.9 ^b^

**Table 4:** Antibacterial activity of the biofunctional textiles measured after three washing cycles (3WC) against four bacterial strains: *Bacillus subtilis*, *Staphylococcus aureus*, *Escherichia coli,* and *Propionibacterium acnes.* Strains in mid-exponential phase were transferred to Erlenmeyers with LB with the respective textile (see Section 4 for details). Samples tested were cotton (CO_3WC_), cotton treated with a biosolution (CO + BS_3WC_) composed by a mixture of propolis (1000 µg mL^−1^) and honey (5%) in water, and cotton treated simultaneously with the BS and potassium alum (CO + BS_PA_3WC_), after three washing cycles. After 24 h incubation at 37 °C, antibacterial activity was determined upon colony-forming units count and results were expressed in growth reduction percentage relative to control (CO_3WC_) and respective standard deviation (SD). Statistical analysis was performed by one-way ANOVA, followed by Tukey’s test for significance. Different letters mean statistically significant (*p* < 0.05) differences between mean values.

Strains	Antibacterial Activity % Growth Reduction	
	CO + BS_3WC_	CO + BS_PA_3WC_
Gram-positive bacteria		
*Bacillus subtilis*	47.1 ± 3.5 ^c,d^	99.8 ± 0.01 ^a^
*Staphylococcus aureus*	86.1 ± 9.8 ^b^	90.1 ± 2.7 ^a,b^
*Propionibacterium acnes*	56.9 ± 9.4 ^c^	54.8 ± 13.3 ^c^
Gram-negative bacterium		
*Escherichia coli*	43.6 ± 0.9 ^d^	13.6 ± 2.1 ^e^

## Data Availability

The data are contained within the article.
